# Factors Affecting the Duration of Nestling Period and Fledging Order in Tengmalm’s Owl (*Aegolius funereus*): Effect of Wing Length and Hatching Sequence

**DOI:** 10.1371/journal.pone.0121641

**Published:** 2015-03-20

**Authors:** Marek Kouba, Luděk Bartoš, Erkki Korpimäki, Markéta Zárybnická

**Affiliations:** 1 Department of Ecology, Faculty of Environmental Sciences, Czech University of Life Sciences Prague, Prague, Czech Republic; 2 Department of Animal Science and Ethology, Faculty of Agrobiology, Food and Natural Resources, Czech University of Life Sciences Prague, Prague, Czech Republic; 3 Department of Ethology, Institute of Animal Science, Prague, Czech Republic; 4 Section of Ecology, Department of Biology, University of Turku, Turku, Finland; University of Lausanne, SWITZERLAND

## Abstract

In altricial birds, the nestling period is an important part of the breeding phase because the juveniles may spend quite a long time in the nest, with associated high energy costs for the parents. The length of the nestling period can be variable and its duration may be influenced by both biotic and abiotic factors; however, studies of this have mostly been undertaken on passerine birds. We studied individual duration of nestling period of 98 Tengmalm’s owl chicks (*Aegolius funereus*) at 27 nests during five breeding seasons using a camera and chip system and radio-telemetry. We found the nestlings stayed in the nest box for 27 – 38 days from hatching (mean ± SD, 32.4 ± 2.2 days). The individual duration of nestling period was negatively related to wing length, but no formally significant effect was found for body weight, sex, prey availability and/or weather conditions. The fledging sequence of individual nestlings was primarily related to hatching order; no relationship with wing length and/or other factors was found in this case. We suggest the length of wing is the most important measure of body condition and individual quality in Tengmalm’s owl young determining the duration of the nestling period. Other differences from passerines (e.g., the lack of effect of weather or prey availability on nestling period) are considered likely to be due to different life-history traits, in particular different food habits and nesting sites and greater risk of nest predation among passerines.

## Introduction

Growth and development rates of juveniles are closely related to levels of parental care and are crucial components of the life-history of every animal species, with fitness benefits to parents provided through increased growth and survival of offspring [1]. During the period of parental investment, parents are expected to care for offspring until the costs of care outweigh the benefits for parents [2]. On the other hand, it is beneficial for the young to receive parental care which exceeds the parental optimum, and thus there arises some conflict of interest between parents and young [3]. The balance of this parent-offspring conflict may influence the time of fledging in altricial bird species. However, the timing of fledging may not necessarily result from conflict, if both parents and young may benefit from earlier fledging (for example when the risk of nest predation is high [4]). Alternatively, fledging could be a result from sibling competition if interests between siblings in a brood are different [5,6].

Several hypotheses have been proposed about the ultimate factors affecting fledging time in altricial birds. According to the threshold size hypothesis (TSH) suggested by Johnson et al. [7], fledging begins when the most well-developed nestlings reach a specific developmental state. Lemel [8] proposed nestling competition hypothesis (NCH) that fledging may be initiated when a smaller and less-competitive nestlings reach same minimum size and leave the nest in order to attract more specific individual attention regarding feeding needs and/or in order to seek food on their own. Finally, kin selection hypothesis (KSH) suggested by Freed [9] predicts that inclusive fitness optimization might prevent the best developed individuals from initiating fledging before their siblings are sufficiently developed, too.

The age at leaving the nest (i.e., fledging) is often associated with attainment of flight and marks a major life-history transition for birds that can be directly affected by morphology [10]. Several studies have shown that nestlings may slow growth and maturation under poor food conditions or experimentally restricted food intake [11–16]. During the early post-hatching period, young altricials are particularly likely to experience nutritional stress because of limitations on the ability of their parents to deliver food [17]. Consequently, food shortage experienced during the energetically demanding nestling period may have a considerable impact on growth and development prior to fledging and on future survival and fitness of altricial birds in the wild [18].

Between species there is a wide variation in the size at the time of fledging as proportion of adult size [19], and increasing nest mortality rate seems to be the one of the most important factors accelerating growth rate of juveniles and thus reducing age at fledging [20]. Other studies have also suggested that growth rates of altricial nestlings are strongly correlated to nest predation rates and nestlings of species under stronger predation pressure remained in the nest for a shorter period, and left the nest at lower body mass relative to adult body mass [21–23]. Overall, the results of various studies of the factors affecting length of nestling period in natural conditions conclude that the most important variables explaining intraspecific variability in time to fledging are: weather conditions, food availability, nestling size and also nest parasitism [7,11,15,24–29]. However, the majority of these studies were performed on passerine birds and none, with the exception of the food-supplementation study by Karell et al. [29] on Ural owls (*Strix uralensis*), have addressed the factors influencing duration of nestling period in diurnal raptors and owls. There is, as a result, very little information available for these predatory species despite the fact that better knowledge of the factors affecting length of the nestling periods in these species may increase our understanding of this important life-history stage in birds of prey.

Tengmalm’s owl (*Aegolius funereus*) is a small nocturnal cavity-nesting owl (male body mass ca. 100 g), living in coniferous forests in the boreal zone and in alpine forests further south in Eurasia [30]. It feeds mainly on small mammals [31–34], and male provides nearly all food to the female from egg laying until she terminates her stay on the nest, and also, thereafter, for the young until independence [31,35–38]. The female incubates the eggs, broods the young, and remains almost continually in the nest cavity until the young are about 3 weeks old [31,39]. Hatching occurs at approximately two-day intervals (at roughly the same intervals as the eggs are laid; [31,40,41]). Therefore, first-hatched chicks are an average one week older than last-hatched chicks [42] which results in a marked size hierarchy among members of the brood.

The individual young stay in the nest for 27–38 days after hatching [31,40,43–45]. In general they leave the nest in the order of hatching one by one at intervals of about one day and fledging period is longer the bigger the brood [31]. Fledglings reach independence 5–9 weeks after fledging [36,37,46,47]. Wing length of nestlings continues to grow with age from hatching until the period after fledging (owlets fledge with incomplete feather growth), while body weight increases only to 3–4 weeks after hatching, and thereafter is essentially stable or may even decline [31,48].

In this paper we examine individual duration of nestling period (i.e., duration of stay of individual nestlings at the nest box from hatching to fledging) of Tengmalm’s owl young and explore factors affecting its length. According to observations from a previous study [31] we predicted (i) that fledgling order of individual nestlings will be closely related to their hatching order. Further, the overall duration of the nestling period in a particular nest will decrease with both (ii) decreasing body weight of nestlings at the end of nestling period and (iii) increasing wing length of nestlings. This should be due to the fact that nestlings start to lose weight 3–4 weeks after hatching till fledgling [31,48], since the flight is easier with less weight. Similarly, individuals with longer wings will be sooner able to leave the nest cavity and fly. Because neither food availability nor weather conditions affected growth rate of both nestlings’ body weight and wing length [48] we further predicted that the duration of individual nestling period will not be related to (iv) food availability and/or (v) weather conditions. Finally, female owls and diurnal raptors are markedly larger than male conspecifics (so called reversed sexual size dimorphism; [30,49]), but the effect of reversed sexual size dimorphism on duration of fledging period remains poorly understood. However, one can predict (vi) that smaller male chicks fledge at a younger age than larger female chicks to escape the dominance of female chicks in competition for food in the nest.

## Materials and Methods

### Study area

The study was conducted over five breeding seasons, in Finland during the breeding season of 2005, and in the Czech Republic during 2006 and from 2010 to 2012. The Finnish site was situated in the Kauhava region of western Finland (63° N, 23° E; 50–110 m a. s. l.), covered ca. 1300 km^2^, and included 450 nest boxes [30,50]. The Czech site (50° N, 13° E; 730–960 m a. s. l.) was situated in the Ore Mountains, covered ca. 70 km^2^, and included 120–170 nest boxes. Nest boxes were square in section and made of wood, with base 18–25 x 18–25 cm, height 40–60 cm and an entrance hole 8–10 cm in diameter.

Information on daily temperature (°C), wind speed (m/s), and precipitation (mm) during the breeding season of Tengmalm’s owl (April—July) were obtained from the Czech Hydrometeorological Institute and the Finnish Meteorological Institute. All data were obtained from weather stations located within the study areas.

### Field procedures

All nest boxes in both study areas and all study years were visited at intervals of 2–3 weeks from early March to July to find nests, and thereafter, nests were checked sufficiently often to know the number of eggs and hatchlings and to determine exact hatching date (± 1 day). The age of the nestlings was in most cases based on the recorded date of hatching. In cases where the exact date of hatching was not recorded, the ages of such nestlings were estimated according to the growth curves (for wing length and body weight) valid for each of the studied populations [30,31,48]. All individuals were weighed and the length of wing was measured at weekly intervals. Duration of stay in the nest box and the date of leaving the nest of all individuals was recorded differently in 2005 in Finland and in 2006 in the Czech Republic, compared to 2010–2012 in the Czech Republic (see below). In total, 98 nestlings from 27 nests were monitored; 32 individuals from 9 nests in Finnish study site, and 66 individuals from 18 nests in Czech site (for details see Table 1).

**Table 1:** Basic breeding data of both studied populations, individual duration of nestling period, and prey availability in both study sites and all study years.

Study site	Year	Basic breeding data					Nestling period duration (days)			Prey availability (prey items per 100 trap nights)
		No. of nests	No. of fledglings	Mean	SD	Range	Mean	SD	Range	
Finland	2005	9	32	3.6	1.6	1–6	32.6	1.6	30–36	13.3
Czech Republic	2006	5	11	2.2	1.2	1–4	33.0	1.2	31–35	0.3
	2010	6	35	5.8	1.7	3–8	31.7	2.3	28–36	10.2
Czech Republic	2011	5	10	2.0	1.1	1–4	34.2	3.1	27–38	0.6
	2012	2	10	5.0	0.0	5	31.5	1.5	30–33	4.9

We defined “nestling period” as the number of days between hatching and leaving the nest (i.e., fledging) for each individual nestling and “fledging period” as the number of days between leaving the nest (i.e., fledging) by the first and the last sibling for each individual brood.

In 2005 and 2006 the date of fledging of all individuals was recorded by using special nest boxes equipped with camera and chip system (see e.g., [51,52]). The equipment consisted of a camera (DECAM), a chip reader device, a movement data-logger, a movement infrared detector (KS96), and infrared lightning (IR diodes, SFH 485–2 880 nm, [53]). All nestlings were marked by chip rings (BR chip ring, BENZING), attached on the foot. A chip aerial affixed by the nest box entrance detected chip rings in its vicinity, i.e., only chip rings of the individuals appearing at or near (up to approximately 5 cm) the entrance of the nest box (the distance between the nest box floor and the bottom of the nest box entrance hole being 25–35 cm). The chip reader recorded the time and the date of detection and the chip code of the ring. The camera was also installed inside the nest box opposite to the entrance. It was triggered by an infrared detector sensitive to movements in the nest box entrance. During the night, the entrance hole of the nest box was illuminated by infrared diodes at the time of the cameras’ making photos. The time of detection was recorded by the movement data-logger and one to three photos were taken for each event. Using this equipment, we were able to record time and date of each nestlings’ fledging event.

In 2010–2012 nestlings were equipped with leg-mount transmitter type PIP4 (Biotrack Ltd., UK) about four days before fledging. For details of tagging the nestlings see Kouba et al. [47,54]. Thereafter, nest boxes were visited at 12–hour-intervals during the night (22:00–04:00) and during daylight (10:00–21:00) until all siblings had fledged and we could determine the exact date of nest box departure. However, in 6 cases (12 nestlings) we could not determine the exact fledging order because these individuals fledged during the same day or night.

Prey availability (small mammals) was assessed by using snap-traps in both study areas in late spring (mid May in the Finnish site, beginning of June in the Czech site); snap-traps were set up in squares (with 10 m spacing). The traps were left out for 3 days and checked daily. In the Finnish study site, the total trapping effort was 1230 trap nights (n = 8 locations); in the Czech study site, the total trapping effort was 1089 trap nights (n = 3 locations). Number of captured mammals per 100 traps nights was calculated for each trapping site. All trapped individuals (n = 166 in the Finnish site, n = 173 in the Czech site) were identified to the species level. For details of prey availability in different study years see Table 1.

Following the method of Hipkiss & Hörnfeldt [55] a 50 μl blood sample was taken from each nestling from the Czech Republic (with the exception of one young; n = 65) by brachial vein puncture under the wing, ca. 14 days after hatching, for molecular sexing. Sex determination of nestlings relied on polymerase chain reaction (PCR) amplification of one intron from the sex chromosome linked *CHD1* gene, which in birds differs in size between the Z and W chromosomes [56]. Males showed only the shorter Z-fragment, while females were characterised by displaying both a 1.2 kb W-specific and a 0.7 kb Z-specific fragment [56].

Owls were trapped, handled, blood sampled and tagged under permit No. 35016/02-OOP/8751/02 and 530/758 R/08-Abt/UL from the Ministry of the Environment of the Czech Republic, were ringed under the Ringing Centre of the National Museum in Prague permit No. 329 and 942, as well as under the Finish Museum of National History (licence No. 524); all efforts were made to minimize suffering.

### Statistical analyses

All data were analysed with the aid of SAS System version 9.3 (SAS Institute Inc.). The analysis was made in two steps. Firstly, to verify that there were no differences in the range of the duration of individual nestling periods between study areas we used a multivariate General Linear Mixed Model (GLMM, PROC MIXED) with individual duration of nestling period and values of fledging order as a dependent variable with area (Finland and the Czech Republic) as fixed effect and individual nest box, study site and season as a random effect. The significance of fixed effect in the GLMM was assessed by the F-test. We used a Tukey-Kramer adjustment for multiple comparisons.

In the second step we tested the associations between (I) the individual duration of nestling period (i.e., duration of stay of individual nestlings at the nest box from hatching to fledging) and other variables and (II) fledging order for individual broods and other variables (fixed and random effects) using a GLMM in both cases. All analyses were designed with individual nesting box and study season as a random effect.

With the exception of individual duration of nestling period (I) and fledging order (II) in particular analyses where these were dependent variables, fixed effects employed within both models were: date and order of hatching, body weight at fledging (g), wing length extrapolated to the age of 30 days from hatching (mm; nestlings were of different ages at time of measurement; in order to be better comparable between individuals wing length was extrapolated by simple proportion to a common date), number of eggs, hatchlings and fledglings from a particular nest box, date and order of fledging, individual duration of nestling period (days), sex of nestlings, mean daily temperature (°C), mean wind speed (m/s), daily precipitation (mm) and type of year (different food availability). Data on weather were log-transformed to normalise the data. All relevant data used in the analyses are presented in the Supporting Information file (S1 Dataset).

We constructed the GLMMs (I and II) by entering first those factors which we expected to have an effect on individual duration of nestling period (nestlings’ wing length and body weight at fledging and sex of individuals—I) and on fledging order (hatching order—II) and then checking both models with addition of the factors which might also affect the result. The significance of each fixed effect in the GLMMs was assessed by the F-test. The effect of sex of nestlings on individual duration of nestling period (I) and fledging order (II) was tested only on Czech subset of the whole dataset (Finnish nestlings were not sexed). If not specifically explained, non-significant factors (P > 0.05) were dropped from both models and will not be mentioned any further. Where appropriate we tested interaction terms. Associations between the dependent variable and fixed effects were estimated by fitting a random coefficient model using PROC MIXED as described by Tao et al. [57]. We calculated predicted values of the dependent variables and plotted them against the fixed effects with predicted regression line.

## Results

The individual duration of nestling period from Finland and the Czech Republic respectively did not differ (mean ± SD; 32.6 ± 1.6 and 32.2 ± 2.4 days; GLMM, F_1, 26.3_ = 0.16, NS). We therefore subsequently pooled data from both areas for further analyses and did not further consider the effect of the area.

Nestlings stayed in the nest box for 27–38 days from hatching (32.4 ± 2.2 days, mean ± SD, n = 98; for details see Table 1). The variation in nestling period of individuals from within a single nest was up to 6 days (with mean and variance 2.7 ± 1.5 days, n = 21; singleton-fledgling nests were excluded). In the Czech Republic male nestlings (n = 37) stayed in the nest box for 32.5 ± 2.6 days and female nestlings (n = 28) for 31.9 ± 2.1 days from hatching on average. The shortest nestling period (27 days) was recorded in poor food year in 2011. This younger individual fledged 6 days earlier than the older one perhaps in order to escape siblicide from the older and bigger sibling. However, the young was found dead less than a day after leaving the nest box and the subsequent autopsy revealed starvation as a case of death [45].

Otherwise, majority of all nestlings (74%) fledged in the order of hatching and fledging period was longer the bigger the brood. Others (14%) fledged sooner or later than the closest sibling and the rest of the individuals (12%) fledged during the same day or night and we could not determine the exact fledging order. Specifically, fledging period was 1.5 hours—15 days long (7.5 ± 4.1 days, n = 21; singleton-fledgling nests were excluded). The shortest period between fledging of the first and last chick was recorded for two-member brood and the longest for a seven-member brood of fledglings, although, there was one (eight-member) brood of fledglings for which the fledging period lasted 14 days.

### Duration of nestling period

**Fig 1:**
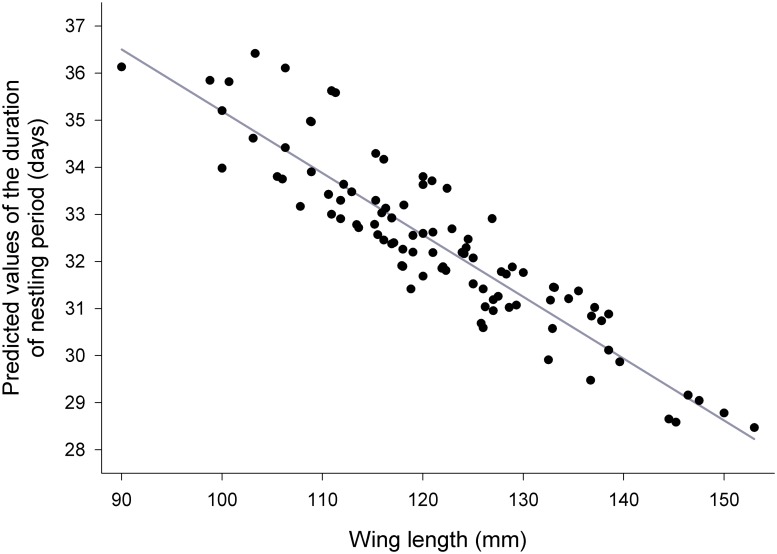
Predicted values of the individual duration of nestling period plotted against the nestlings’ wing length extrapolated to the age of 30 days from hatching.

The results of the GLMM for the individual duration of nestling period (I) revealed that it was dependent on nestlings’ wing length extrapolated to the age of 30 days from hatching only (F_1, 89.5_ = 49.70, P < 0.0001). Length of nestling period was shorter for chicks showing the most rapid rate of increase in wing length (Fig. 1) who thus reached the critical wing length required for successful fledging more quickly. Neither brood size at hatching or fledging, nestlings’ body weight, sex of individuals, type of year (prey availability), weather conditions (mean daily temperature, mean wind speed, and daily precipitation) nor interactions between wing length and nestlings’ body weight, sex and wing length, brood size and wing length, brood size and nestlings’ body weight were significantly related to the individual duration of nestling period.

### Fledging order

**Fig 2:**
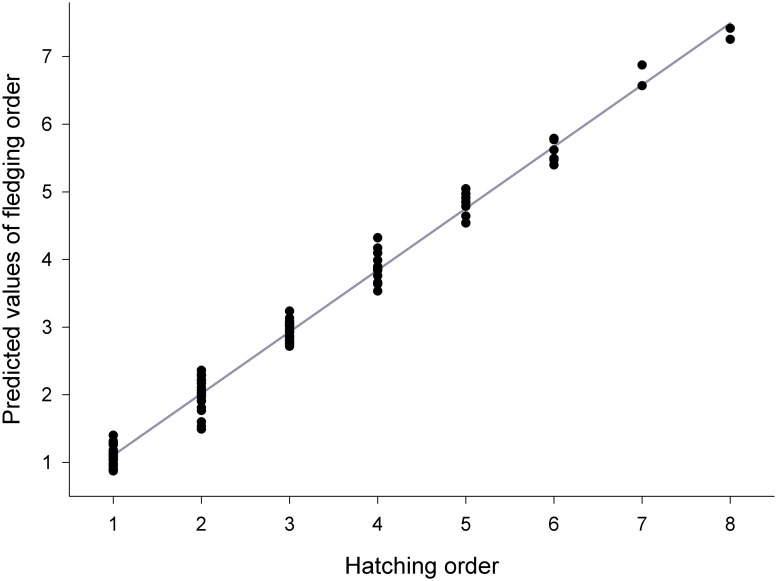
Predicted values of the fledging order plotted against the order of hatching within individual broods.

The results of the GLMM for the fledging order (II) of individual nestlings from individual broods revealed that fledging sequence was dependent on hatching order of individuals only (F_1, 98_ = 975.67, P < 0.0001). Order of fledging correlated closely with order of hatching (Fig. 2). Wing length of nestlings extrapolated to the age of 30 days from hatching, sex of individuals and/or other factors were not significantly related to the order of fledging.

## Discussion

We found the order of fledging was dependent on hatching order as we expected (i). This is in accordance with previously published information on Tengmalm’s owl young [31]. We did not find any obvious relationship between fledging order and wing length although this has been observed in several passerines [4,7,27,58]. This could be because of short or very short intervals between hatching of individuals in passerine species compared with approximately 2-day intervals in Tengmalm’s owl which results in a marked hatching asynchrony in owls [42], and variations in individual growth rate which are much more marked in passerines.

In Tengmalm’s owls differences in growth rates between individuals are quite small after hatching and nestlings grow at approximately same rate up to 3 weeks after hatching of the first individual because up to this time the female is present in the nest box and could divide the prey delivered by the male relatively evenly between the chicks [31,48]. Thereafter, and once the young are able to thermoregulate effectively on their own, the female leaves the box; from this point sibling competition may become more marked and differences in growth rates between individuals may increase [59]. During later part of the nestling period the wing lengths of young reaching a given age can differ by up to 1 cm (Kouba & Zárybnická unpublished data); this may be enough to result in significant differences in overall duration of the nestling period in different individual chicks but probably not large enough to significantly affect the order of fledging. It seems the 2-day gap between hatching is so long that wing lengths of younger siblings rarely overtake those of older ones. We did note however that 14% of chicks fledged sooner or later than the closest sibling and the exact fledging order of 12% could not be determined. The reason for chicks choosing to fledge out of sequence with strict hatching order may be in order to increase the chances of being fed, by being outside the nest, if older siblings are monopolizing access for food provided by the parents inside the nest box [27].

The reason why the order of fledging closely followed the order of hatching could be connected also with social dominance status within particular broods. The largest and most dominant nestling in the nest benefits from maintaining its prime feeding position, but in the course of time, the other nestlings catch up in body size and weight (although the wing length continues to growth after fledging, nestlings reach an asymptote in body size and weight within the nest, reaching full adult body size before fledging with the exception of wing and tail length). The prime position of the dominant nestling is challenged more and more and to ensure its prime position, the largest nestling is forced to leave the nest, because parents tend to prefer feeding fledglings rather than nestlings [6,58].

Wing length appeared to be the single and most powerful predictor for assessing the time of leaving the nest by Tengmalm’s owl nestlings with the nestling period decreasing with increasing rate of increasing wing length (prediction iii). Miller [10] showed that there could be high plasticity in wing growth between individuals and that wing area had a direct relationship to fledging time so that individuals with larger wings fledged earlier. Such individuals are most probably better able to fly [60] and thus, fledged earlier compared to those with shorter (or smaller) wings, as has also been reported in several small passerine species such as the Pied flycatcher (*Ficedula hypoleuca*), the Tree swallow (*Tachycineta bicolor*), the House wren (*Troglodytes aedon*) and the Great tit (*Parus major*) [4,6,7,58], respectively. Thus, these results seem to be quite similar across different bird taxa.

Contrary to our second prediction (ii) we found no relationship between nestling body weight before fledging and the duration of nestling period, such that chicks which lose weight more rapidly are fledging earlier. This result was found despite the fact that Tengmalm’s owl nestlings start to lose weight approximately 26 days after hatching [31,48] and similar relationship (weight losses with the approaching end of nestling period) was observed also in Tropical Screech owl (*Megascops choliba*) by Trent [61]. We suggest the loss of weight in owlets at the end of the nesting period could be an adaptation for better flying ability of fledglings which is in accordance with findings by Wright et al. [62] that nestlings of the Common swift (*A*. *apus*) are able to adjust body mass so that they always fledge with aerodynamically appropriate wing loadings. However, at the end of nestling period, wing length of nestlings seems to be much more important for time of fledging and ability to fly than their body weight. This could be applied for Tengmalm’s and Pygmy owls (*Glaucidium passerinum*) in particular whose fledglings are able to fly when leaving the nest compared to other European owls which leave the nest when they are still flightless [41,43]. We suggest that above stated facts regarding fledging process in owls could also explain differences between our findings and those of Karell et al. [29] who reported that supplementary-fed Ural owl nestlings reached higher asymptotic mass which enabled them to fledge earlier than non-supplemented individuals suggesting that nestling growth is food-limited under natural conditions. Karell et al. [29] also reported that age at fledging was advanced in nests with high numbers of blood-sucking blackflies (Diptera, Simuliidae), and thus, a benefit from early fledging appears to be also the reduction of nestlings exposure to parasites, however, we do not have any relevant data to report this in the Tengmalm’s owl.

We found no obvious effect of biotic and abiotic factors on the duration of nestling period. Specifically, there was no relationship between abundance of main food in spring (prediction iv) or weather conditions (v) and the individual duration of nestling period. We suggest that although abundance of main foods is the most important factor influencing timing of egg-laying, clutch size, breeding success [30,32,50,63] and post-fledging behaviour of offspring in Tengmalm’s owl [47,54], it is not decisive in the case of length of nestling period. Although studies have recorded prolonged nestling phases in other species due to lower food availability (primarily caused by harsh weather conditions), we suggest our finding could reflect differences between life-history traits of studied species regarding nesting places and food habits. Tengmalm’s owl subsists mainly on small mammals [31–33,64] and is a cavity nester [43]. By contrast, those species for which an effect of weather was recorded on duration of the nestling period [11,25,26,65] were insectivorous and piscivorous species nesting mostly on the ground. The cavity environment is more stable regarding weather conditions, thus there is likely to be a lesser need to prolong the nestling period due to higher thermoregulatory costs, for example. Moreover, the availability of small mammal prey is not so strongly influenced by weather conditions as is the availability of insect and marine foods.

Finally, we found no obvious effect of sex of nestlings on fledging order or the duration of nestling period (vi). This finding is further supported by the fact that no obvious difference was detected in wing length between sexes as revealed by lack of significance when an interaction between sex and wing length entered the GLMMs. We suggest the inter-individual differences in nestling body size and weight related to sex of individuals which are caused by reversed sexual size dimorphism [30,49] are most likely not large enough at this stage of ontogeny to affect fledging order or the duration of nestling period in the Tengmalm’s owl.

As far as we know, apart from Karell et al. [29], this is the only study on any owl or diurnal raptor species evaluating factors affecting duration of nestling period. Compared to small passerines [22,23] or Mourning doves (*Zenaida macroura*) [10] which are evolutionary selected for early fledging (fledging at lower body size and weight contrary to adults) due to exposure to high nest predation rates during whole nesting period [66], Tengmalm’s owl does not seem to be evolutionary selected in the same way. Their nests are mostly predated during egg-laying or incubation period (77% of nests in the Czech study site which did suffer predation were predated during this earlier period) while predation is rare during late nestling phase (M. Zárybnická, J. Riegert & M. Kouba unpublished data). The same was reported from Norway and Finland that the vast majority of all predation events occurred during the early breeding phase [67,68]. Moreover, the Tengmalm’s owl young fledged with body weight equal to that of adults compared to species where nestlings left the nest at lower body weight relative to that of their adults [21–23]. Thus, it seems life-history traits regarding selective pressures for fledging are different among these species.

We conclude that our findings show no obvious effect of main food abundance, brood size, sex of nestlings, weather condition or body weight of nestlings on the duration of nestling period, while it strongly highlights the influence of wing length (as a measure of body condition and individual quality; [16,69,70]) on the duration of individual nestling period. It supports the threshold size hypothesis (TSH; [7]) that fledging begins when nestlings reach a specific developmental state, a hypothesis also supported by other studies [4,27,58].

## Supporting Information

S1 Dataset Relevant data used in the analyses (GLMM I and GLMM II). (XLS)
